# Supplementary material for: Multimodal imaging and functional analysis of the chick NMDA retinal damage model
Source: PLoS One. 2021 Sep 7;16(9):e0257148. doi: 10.1371/journal.pone.0257148 (PMC8423281; doi:10.1371/journal.pone.0257148)
Supplement: S1 File — (ZIP) [file pone.0257148.s004.zip › SD-OCT/C200727/009/D8-PWk2/C200727-009_OD_V_14X14_0_REGAVG0000042/Manual_PreDefineFiveByFiveGridRectangularRetina_1.pdf]

Five By Five Grid Template Report

Patient ID	C200727	Name	See InVivoVue
Exam Date	See InVivoVue	Exam Time	See InVivoVue
Eye	OD	Averaged	N/A
Depth (mm)	1.65	Depth Samples	1024
Pitch (um/pix)	35.000 x 35.000 x 1.608	Refractive Index	See InVivoVue
Scan Type	Rectangular 14.000mm x 14.000mm x 1.647mm , 400 x 400 x 1024		

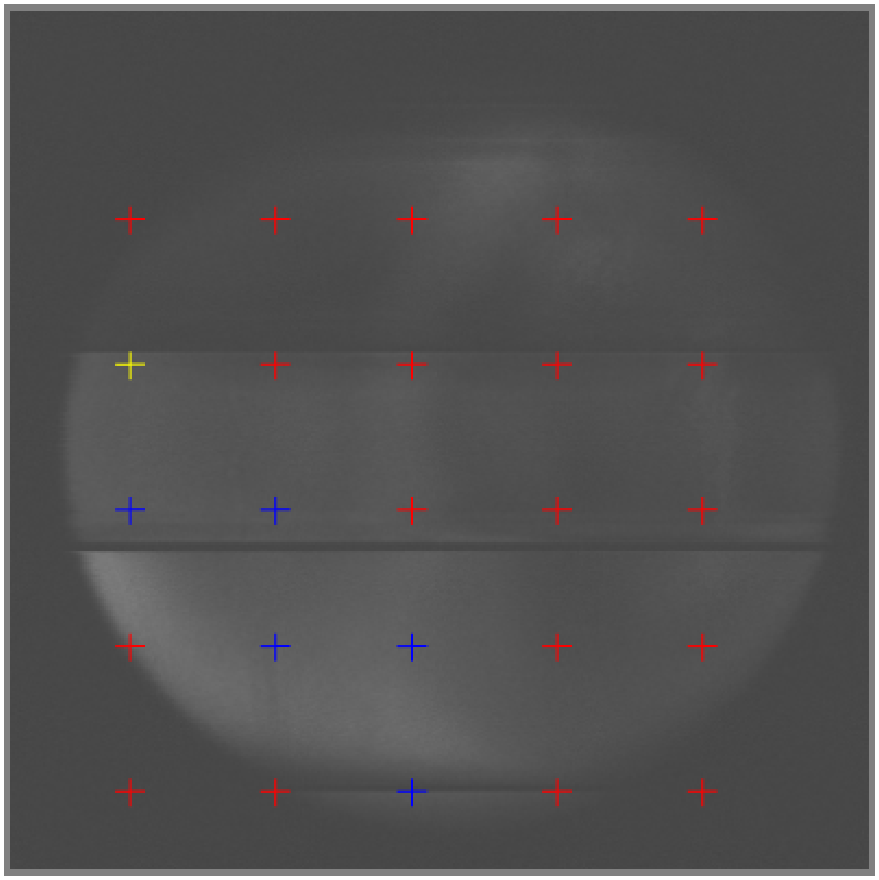

Thickness Metrics

Analysis	Average(mm)	Minimum(mm)	Maximum(mm)	STDEV(mm)
IRNFL - RPE	0.2825	0.2634	0.2939	0.0122
IRNFL - ORNFL	0.0472	0.0175	0.0811	0.0230
ORNFL - OPL	0.1623	0.1404	0.2047	0.0261
OPL - ETPRS	0.0554	0.0518	0.0587	0.0029
